# Predictive value of baseline metabolic tumor burden on ^18^F-FDG PET/CT for brain metastases in patients with locally advanced non-small-cell lung cancer

**DOI:** 10.3389/fonc.2022.1029684

**Published:** 2022-10-26

**Authors:** Jingjie Shang, Huimin You, Chenchen Dong, Yingxin Li, Yong Cheng, Yongjin Tang, Bin Guo, Jian Gong, Xueying Ling, Hao Xu

**Affiliations:** ^1^ Department of Nuclear Medicine and Positron Emission Tomography (PET)/Computed Tomography (CT)-Magnetic Resonance Imaging (MRI) Center, The First Affiliated Hospital of Jinan University, Guangzhou, China; ^2^ Department of Endocrinology, The Fifth Affiliated Hospital of GuangZhou Medical University, Guangzhou, China

**Keywords:** non-small-cell lung cancer, brain metastases, PET/CT, metabolic tumor volume, predictive value

## Abstract

**Objectives:**

Brain metastases (BMs) are a major cause leading to the failure of treatment management for non-small-cell lung cancer (NSCLC) patients. The purpose of this study was to evaluate the predictive value of baseline metabolic tumor burden on ^18^F-FDG PET/CT measured with metabolic tumor volume (MTV) and total lesion glycolysis (TLG) for brain metastases (BMs) development in patients with locally advanced non-small-cell lung cancer (NSCLC) after treatment.

**Methods:**

Forty-seven patients with stage IIB-IIIC NSCLC who underwent baseline ^18^F-FDG PET/CT examinations were retrospectively reviewed. The maximum standardized uptake value (SUV_max_), MTV, and TLG of the primary tumor (SUV_maxT_, MTV_T_, and TLG_T_), metastatic lymph nodes (SUV_maxN_, MTV_N_, and TLG_N_), and whole-body tumors (SUV_maxWB_, MTV_WB_, and TLG_WB_) were measured. The optimal cut-off values of PET parameters to predict brain metastasis-free survival were obtained using Receiver operating characteristic (ROC) analysis, and the predictive value of clinical variables and PET parameters were evaluated using Cox proportional hazards regression analysis.

**Results:**

The median follow-up duration was 25.0 months for surviving patients, and 13 patients (27.7%) developed BM. The optimal cut-off values were 21.1 mL and 150.0 g for MTV_T_ and TLG_T_, 20.0, 10.9 mL and 55.6 g for SUV_maxN_, MTV_N_ and TLG_N_, and 27.9, 27.4 mL and 161.0 g for SUV_maxWB_, MTV_WB_ and TLG_WB_, respectively. In the Cox proportional hazards models, the risk of BM was significantly associated with MTV_N_ and MTV_WB_ or TLG_N_ and TLG_WB_ after adjusting for histological cell type, N stage, SUV_maxN_, and SUV_maxWB_.

**Conclusions:**

Baseline metabolic tumor burden (MTV and TLG) evaluated from the level of metastatic lymph nodes and whole-body tumors are significant predictive factors for BM development in patients with locally advanced NSCLC.

## Introduction

Brain metastases (BMs) are the most frequent types of failure in patients with locally advanced non-small-cell lung cancer (NSCLC) (1–3), which is related to significant neurocognitive impairment, life quality deficits, poor prognosis, and high mortality (4). Previous studies have shown that prophylactic cranial irradiation (PCI) can delay the onset or reduce the occurrence of BM in locally advanced NSCLC, but failed to demonstrate a significant improvement in overall survival (OS) (5, 6), which suggests that such therapy may not be appropriate for all NSCLC patients. Therefore, identifying potential patients who may benefit from PCI is important to reduce brain relapses and improve their outcomes.

BM develops following the haematogenous spread of tumor cells to the brain microvasculature. Initially, cancerous cells need to separate from the primary tumor, infiltrate into the surrounding tissues, then enter the vasculature and lymphatic system (7). The assessment of tumor aggressiveness is helpful to predict the occurrence of BM (8). For patients with locally advanced NSCLC, the primary tumor status (T), especially the regional lymph node involvement status (N), can reflect the invasiveness and growth characteristics of the tumors (9). However, the conventional T and N stages are based on the size and location of the tumor and could not comprehensively reflect aggressiveness, and tumor-specific factors vary even among patients within the same disease stage, creating a heterogeneous subgroup concerning the prognosis (10). Therefore, BM predictors based on tumor biology are needed to reflect the biological characterization of aggressiveness.

^18^F-FDG PET/CT is a well-established molecular imaging technology that enables non-invasive quantification of tumor biological characteristics. It is now routinely used for diagnosis, staging, outcome prediction, and response evaluation for NSCLC patients (11). Several studies have reported that the maximum standardized uptake value (SUV_max_), a semiquantitative index that reflects tumor metabolic activity, is a significant prognostic imaging marker for NSCLC patients, but it cannot represent uptake for the entire tumor mass (12). More recently, there has been a growing recognition of the metabolic tumor volume (MTV) and total lesion glycolysis (TLG), the volumetric PET parameters which have been explored as measures of metabolic tumor burden, as promising quantitative PET indices. Studies have shown that MTV and TLG predict prognosis better than SUV_max_ and tumor stage for NSCLC (13–15). Nevertheless, whether volumetric PET parameters could predict BM development in NSCLC patients has not been reported.

Therefore, the aim of our current study was to evaluate the predictive value of baseline MTV and TLG for BM development in locally advanced NSCLC patients and compare them with other predictors.

## Materials and methods

### Patients

All patients with newly diagnosed stage IIB-IIIC NSCLC who received baseline ^18^F-FDG PET/CT examination between November 2012 and November 2020 were retrospectively reviewed. Those patients who had negative baseline brain magnetic resonance imaging (MRI) results, had no history of other prior malignancies, and had complete follow-up information were eligible for the study. The patient’s demographic and clinic pathological variables at the time of diagnosis were recorded, which included age, sex, histology, smoking status, and tumor stage.

Patients were followed up every 3 months for 2 years, and then every 6 months thereafter. The follow-up evaluations consisted of medical history, physical examination, thoracic CT, abdominal ultrasound, and other necessary examinations as clinically indicated. Contrast-enhanced brain MRI was performed if patients had suspicious symptoms or disease progression. The physician determined disease progression based on available information, including clinical assessments, radiologic examinations, and pathology reports. This study was approved by our institutional review board (Number: KY-2021-075) and complied with national legislation and the guidelines of the Declaration of Helsinki. Due to the retrospective nature of this study, informed consent was not required.

### PET/CT acquisition

All examinations were performed using a standard protocol on GE Discovery PET/CT 690 scanner. Patients were required to fast for at least 6 h with serum glucose concentrations < 200 mg/dL before the examination. Imaging was obtained 50 to 70 min after intravenous injection of ^18^F-FDG (0.08-0.10 mCi/kg). A whole-body unenhanced CT scan was firstly performed with 130 kV, 100-180 mA modulated based on the patient’s body weight. After the whole-body CT scanning, PET data were acquired using the time-of-flight (TOF) technology. The PET images were attenuated correction in terms of CT data and reconstructed using the TOF together with point spread function (PSF) technology.

### Measurement of PET parameters

**Figure 1:**
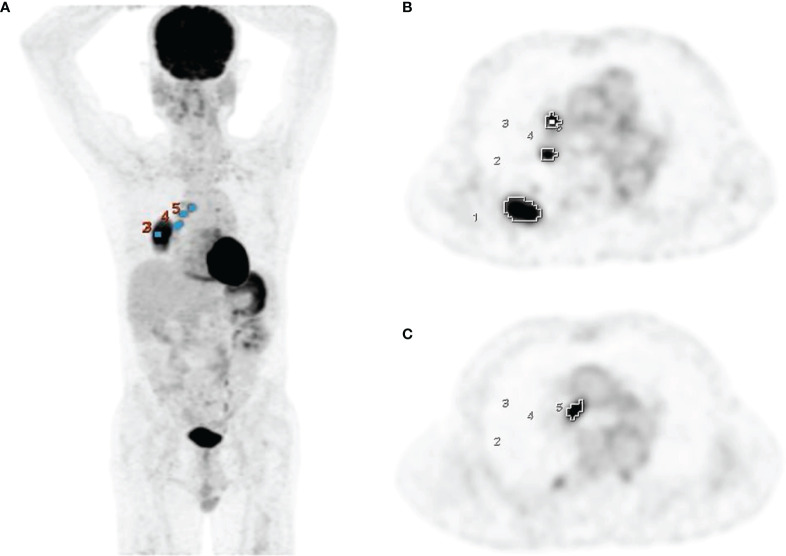
Measurement of PET parameters using PET VCAR on PET coronal **(A)** and axial images **(B, C)** in a 69-year-old man with adenocarcinoma.

pt?>PET volume computer-assisted reading (PET VCAR), an automated segmentation system in the GE Advantage Workstation, was used to calculate the PET parameters of tumors. PET VCAR can separate the target tumor from the background tissue based on a threshold value calculated by an iterative adaptive segmentation algorithm, and our previous studies have described the detailed instructions of this software (16, 17). By drawing a volume of interest around the tumor, the SUV_max_, MTV, and TLG of the entire tumor could be measured automatically (**Figure 1**). Sometimes needed to adjust manually the estimated tumor surface to include the margins of the entire tumor within the volume of interest. All hypermetabolic metastatic lesions were selected for analysis, while hypermetabolic foci explained by physiological activity or inflammation were excluded. The SUV_max_, MTV, and TLG of the primary tumor, metastatic lymph nodes, and whole-body tumors were measured and denoted as SUV_maxT_, MTV_T_, TLG_T_, SUV_maxN_, MTV_N_, TLG_N_, SUV_maxWB_, MTV_WB_, and TLG_WB_, respectively.

### Statistical analysis

Brain metastasis-free survival (BMFS), the time from initial therapy to when the BM was confirmed by a brain MRI scan, was recorded as the primary endpoint. OS, the time from treatment initiation to death or final follow-up, was recorded as the secondary endpoint. The Mann-Whitney U test was used to analyze the difference in PET parameter values between the BM-negative and BM-positive groups, and *p* < 0.05 was considered significant. Significant parameters related to BM were further analyzed using receiver operating characteristic (ROC) analysis to determine the optimal cut-off values to predict BMFS. Patients were dichotomized based on these optimal cut-off values. The BMFS and OS curves of different groups were estimated using the Kaplan–Meier method and compared by the log-rank test, and *p* < 0.05 was considered significant. Risk factors for BM development, including various PET parameters and other clinical variables, were identified using univariate and multivariate Cox regression analyses. Each factor whose *p* < 0.1 in the univariate analysis was further analyzed by the multivariate analysis. Two separate multivariate models were used to analyze MTV and TLG because there was multicollinearity between them. All data were processed with SPSS software.

## Results

### Patient characteristics

Two hundred and three patients with newly diagnosed stage II B-IIIC NSCLC were retrospectively reviewed. We excluded 128 patients for whom the clinical data were incomplete or lost to follow-up, 15 patients who had a history of other prior malignancy, and 13 patients who were treated with curative surgery. Finally, a total of 47 patients (27 men and 20 women; mean age 64 years, range, 42-85 years) were enrolled in this study, of which 25 were adenocarcinoma, 17 were squamous cell carcinoma and 5 were large cell carcinoma. The tumor stage was IIB in 6 patients, IIIA in 19, IIIB in 12 and IIIC in 10 patients. The treatment approach consisted of chemoradiotherapy (n = 29) and chemotherapy or targeted therapy (n = 18), which was performed according to each patient’s situation and the corresponding physician’s decision. The chemotherapy regimens included cisplatin- or carboplatin-based regimens in combination with gemcitabine, pemetrexed, paclitaxel, or docetaxel. The targeted therapies included gefitinib, osimertinib, or anlotinib. The total dose of radiotherapy varied from 40 Gy in 20 fractions to 70 Gy in 35 fractions, with a mean prescribed dose of 57.1 ± 10.6 Gy. The patient’s demographics and clinical characteristics are summarized in **Table 1**.

**Table 1:** Demographic and clinical characteristics of the patients.

Characteristic	N (%)
Age	
Median (range)	64 (42-85)
Sex	
male	27 (57.4%)
female	20 (42.6%)
Smoking history	
never	38 (80.9%)
ever/current	9 (19.1%)
Histology	
Adenocarcinoma	25 (53.2%)
Squamous cell carcinoma	17 (36.2%)
Large cell carcinoma	5 (10.6%)
T stage	
1	19 (40.4%)
2	16 (34.0%)
3	5 (10.6%)
4	7 (14.9%)
N stage	
1	6 (12.8%)
2	21 (44.7%)
3	20 (42.5%)
TNM stage (AJCC 8th edition)	
IIB	6 (12.8%)
IIIA	19 (40.4%)
IIIB	12 (25.5%)
IIIC	10 (21.3%)
Primary treatment	
chemoradiotherapy	29 (61.7%)
chemotherapy or targeted therapy	18 (38.3%)

### BMFS and OS analysis

The median follow-up duration among survivors was 25.0 months, with a range of 3.5-105.9 months. At the time of analysis, 21 (44.7%) patients were still alive and 26 (55.3%) patients had died. Overall, 40 patients (85.1%) experienced disease progression: 18 patients (45.0%) had local and/or locoregional recurrence, 9 (22.5%) patients had distant metastases, and 13 (32.5%) patients had both local and/or locoregional and distant recurrence. Thirteen patients (27.7%) experienced BM; of these, 8 patients (61.5%) experienced BM as the first relapse site, and 5 patients (38.5%) developed BM after extracranial disease progression. The median time of BM diagnosis after treatment (BMFS) was 14.3 months (range, 4.1-50.7 months), and the 1-, 3-, and 5-year cumulative rates of BM were 11.5%, 28.4% and 39.8%, respectively. OS was significantly lower among those patients with BM positivity compared with patients without BM positivity (*p* = 0.01).

### PET parameters and ROC analysis

**Figure 2:**
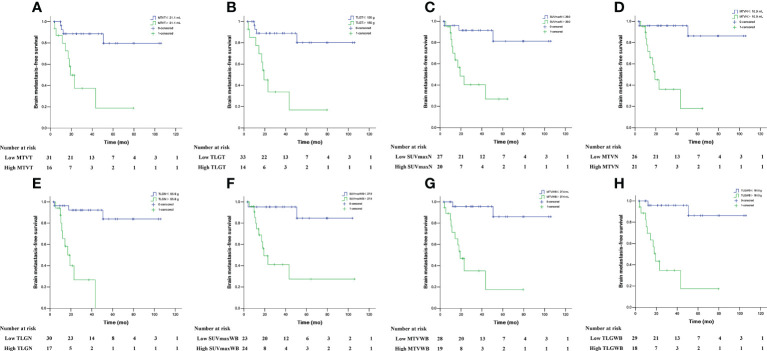
Brain metastasis-free survival in locally advanced NSCLC patients stratified by MTV_T_ **(A)**, TLG_T_ **(B)**, SUV_maxN_ **(C)**, MTV_N_ **(D)**, TLG_N_ **(E)**, SUV_maxWB_ **(F)**, MTV_WB_ **(G)**, and TLG_WB_ **(H)**.

The descriptions of the mean and optimal cut-off values of various parameters as determined by ROC curves are provided in **Table 2**. The differences in SUV_maxT_ between the BM-negative and BM-positive groups were not statistically significant (*p*> 0.05). Conversely, MTV_T_, TLG_T_, SUV_maxN_, MTV_N_, TLG_N_, SUV_maxWB_, MTV_WB_, and TLG_WB_ were significantly different between groups (*p*< 0.05). Furthermore, for 40 patients who experienced disease progression, the differences in MTV_T_, TLG_T_, SUV_maxN_, MTV_N_, TLG_N_, MTV_WB_, and TLG_WB_ between the BM-negative and BM-positive groups were also statistically significant (*p*< 0.05). The optimal cut-off values for BMFS derived from the AUC data were 21.1 mL and 150.0 g for MTV_T_ and TLG_T_, 20.0, 10.9 mL and 55.6 g for SUV_maxN_, MTV_N_ and TLG_N_, and 27.9, 27.4 mL and 161.0 g for SUV_maxWB_, MTV_WB_ and TLG_WB_, respectively. Using these derived optimal cut-off points, the Kaplan–Meier curves of BMFS are shown in **Figure 2**. Patients with high MTV and TLG at the primary tumor, metastatic lymph nodes, and whole-body tumor levels had significantly shorter BMFS (*p*< 0.05).

**Table 2:** The description of mean values and the optimal cutoff values of PET parameters.

Parameter	Total (n=47)	BM positive (n=13)	BM negative^a^ (n=34)	BM negative^b^ (n=27)	AUC (95% CI)	cutoff
SUV_maxT_	13.5 ± 6.7	14.5 ± 4.6	13.2 ± 7.3	13.9 ± 7.3	–	–
MTV_T_ (mL)	28.7 ± 45.5	49.3 ± 65.3	20.8 ± 33.1^*^	24.2 ± 36.5^*^	0.770 (0.631 - 0.910)	21.1
TLG_T_ (g)	229.9 ± 471.4	423.6 ± 672.3	155.8 ± 353.6^*^	187.4 ± 391.9^*^	0.773 (0.624 - 0.921)	150.0
SUV_maxN_	21.2 ± 17.0	28.3 ± 14.5	18.5 ± 17.3^*^	19.7 ± 17.2^*^	0.730 (0.586 - 0.873)	20.0
MTV_N_ (mL)	16.8 ± 31.4	37.5 ± 53.9	8.7 ± 9.3^*^	9.3 ± 9.5^*^	0.829 (0.706 - 0.952)	10.9
TLG_N_ (g)	83.1 ± 204.5	199.0 ± 362.1	38.9 ± 57.5^*^	41.7 ± 61.4^*^	0.813 (0.679 - 0.948)	55.6
SUV_maxWB_	35.8 ± 19.0	42.9 ± 15.2	31.6± 19.6^*^	33.6 ± 18.2	0.708 (0.557 - 0.859)	27.9
MTV_WB_ (mL)	45.5 ± 55.6	86.9 ± 77.2	29.6 ± 34.8^*^	33.5 ± 38.0^*^	0.826 (0.693 - 0.958)	27.4
TLG_WB_ (g)	313.0 ± 512.2	622.5 ± 703.3	194.7 ± 365.6^*^	229.2 ± 403.7^*^	0.821 (0.682 - 0.960)	161.0

Values are presented as number or Mean ± SD.

^a^Patients without BM from the whole study population.

^b^Patients without BM from all recurrences.

Compared the difference in PET parameter values between the BM-positive and BM-negative groups (Mann–Whitney U test).

^*^P <0.05.

### Risk factors of BM

The univariate and multivariate analysis results are summarized in **Table 3**. In univariate analysis, adenocarcinoma histology, high T stage (T2-4), N stage (N3), MTV_T_> 21.1 mL, TLG_T_> 150.0 g, SUV_maxN_ > 20.0, MTV_N_ > 10.9 mL, TLG_N_ > 55.6_ g_, SUV_maxWB_ > 27.9, MTV_WB_ > 27.4 mL, and TLG_WB_ > 161.0_ g_ (all *p*< 0.05) were significantly related to an increased risk of developing brain relapses. Multivariate analysis showed high MTV_N_ (HR = 6.241; 95% CI, 1.037-37.559; *p* = 0.046) and high MTV_WB_ (HR = 6.936; 95% CI, 1.185-40.604; *p* = 0.032) remained risk factors for BM after adjusting for histology, T stage, N stage, SUV_maxN_, and SUV_maxWB_. Furthermore, high TLG_N_ (HR = 5.550; 95% CI, 0.966-31.900; *p* = 0.055) and high TLG_WB_ (HR = 6.491; 95% CI, 1.029-40.961; *p* = 0.047) also remained risk factors for BM after adjusting for histology, T stage, N stage, SUV_maxN_, and SUV_maxWB_.

**Table 3:** The risk factors of brain metastases development.

factor	Univariate analysis			Multivariate analysis		
	HR	95% CI	*p* value	HR	95% CI	*p* value
Age (≤60 VS >60)	0.857	0.279-2.630	0.788			
Sex (female VS male)	0.905	0.299-2.744	0.860			
Smoking (never VS ever/current)	1.467	0.490-4.390	0.493			
Histology (Squamous cell carcinoma VS adenocarcinoma)	5.715	1.260-25.925	0.024			
T stage (T1 VS T2-4)	5.096	1.115-23.289	0.036			
N stage (N1-2 VS N3)	3.905	1.228-12.423	0.021			
TNM stage (IIB-IIIA VS IIIB-C)	2.860	0.916-8.926	0.070			
MTV_T_ (≤21.1 mL VS >21.1 mL)	6.442	1.908-21.751	0.002			
TLG_T_ (≤150.0 g VS >150.0 g)	7.232	2.161-24.199	0.001			
SUV_maxN_ (≤20.0 VS >20.0)	7.379	1.994-27.305	0.003			
MTV_N_ (≤10.9 mL VS >10.9 mL)	13.935	2.913-66.626	0.001	6.241	1.037-37.559	0.046^a^
TLG_N_ (≤55.6 g VS >55.6 g)	17.203	3.573-82.830	<0.001	5.550	0.966-31.900	0.055^b^
SUV_maxWB_ (≤27.9 VS >27.9)	10.095	2.714-46.885	0.003			
MTV_WB_ (≤27.4 mL VS >27.4 mL)	14.703	3.089-69.988	0.001	6.936	1.185-40.604	0.032^a^
TLG_WB_ (≤161.0 g VS >161.0 g)	15.486	3.288-72.940	0.001	6.491	1.029-40.961	0.047^b^

^a^ MTV model, ^b^ TLG model.

## Discussion

The present study indicates that the pretreatment metabolic tumor burden of the primary tumor, metastatic lymph nodes, and whole-body tumors as measured by MTV and TLG has a high potential value in predicting BM development in locally advanced NSCLC. Patients with high MTV_T_, TLG_T_, MTV_N_, TLG_N_, MTV_WB_ and TLG_WB_ were associated with shorter median BMFS. Moreover, the multivariate analysis demonstrated that MTV_N_ and MTV_WB_ or TLG_N_ and TLG_WB_ were significant predictors of an increased risk of relapse with BM, even after adjusting for well-known clinicopathological predictive factors in patients with NSCLC.

BM is a common type of complication in patients with NSCLC, both in early and locally advanced stages after treatment with complete surgical resection and chemoradiotherapy. The occurrence rates of BM in locally advanced NSCLC patients were 25% to 55%, with a median BMFS in the range of 9.0-16.0 months in previous studies (1–3). In most of these patients, BM occurred within 3 years of diagnosis. The results of our study are similar to these observations, with an overall rate of BM of 27.7%, a median BMFS of 14.3 months, and a 3-year actuarial incidence of BM of 28.4%. Furthermore, similar to previous studies (18), we found that the OS of the BM-positive group was significantly lower than that of the BM-negative group.

Several studies have reported that the higher T stages and especially higher N stages of NSCLC were associated with an increased occurrence rate of BM (19–22). In the study by Bajard et al. (19), researchers found that T4 and N2-3 were risk factors for BM development in 305 patients with stage I-III NSCLC. However, with disease development, the accuracy of the T and N stages as a surrogate for tumor burden breaks down due to a wide spectrum of disease severities represented by only a few different stages (10). Some researchers, therefore, proposed using the number or size of metastatic lymph nodes or the ratio of metastatic to examined lymph nodes to predict BM development (23, 24). Ding et al. (24) analyzed 217 patients with stage IIIA-N2 NSCLC and found that the number of metastatic mediastinal lymph nodes ≥ 3 and the ratio of metastatic to examined lymph nodes ≥ 30% were significantly correlated with a risk of BM development.

The volumetric PET parameters MTV and TLG can provide a more complete estimation of the tumor burden and biological aggressiveness. Previous studies have demonstrated that metabolic tumor burden is a significant and independent predictive factor for progression in patients with NSCLC (13–15). However, none of these specifically focused on the development of BM. In the present study, we conducted a comprehensive evaluation of volumetric PET parameters at different levels and estimated optimal cut-off points to propose clinically predictive markers for BM. We found that MTV and TLG of the primary tumor, metastatic lymph nodes, and whole-body tumors were significantly different between the BM-negative and BM-positive groups in all patients and all recurrences. Furthermore, the multivariate analysis showed that MTV_N_, MTV_WB_, and TLG_WB_ were independent predictors of BM (all *p* < 0.05), even after adjusting for histology, T stage, N stage, SUV_maxN_, and SUV_maxWB_. TLG_N_ also showed a trend as an independent predictor for BM development (*p* = 0.055). More accurate risk stratification may aid clinician and patient decision-making for optimal treatment choices and better outcome prediction. Patients with high baseline MTV or TLG should be closely monitored after treatment and potentially enrolled in novel treatment approaches. Therefore, incorporating metabolic tumor burden in therapeutic regimens could help select patient groups that would most benefit from PCI therapies.

The current study showed that, unlike volumetric PET parameters, the SUV_max_ of the primary tumor was not significantly different between the BM-negative and BM-positive groups. Furthermore, the multivariate analysis showed that SUV_maxN_ and SUV_maxWB_ were not significant predictors of an increased risk of relapse with BM. This may be partly because SUV_max_ only represents the measurement from a single spot of the most hypermetabolic area of a tumor mass and does not reflect accurately the metabolic activity of the whole tumor (12). Indeed, multiple studies evaluating the prognostic value of MTV and TLG, which reflect the whole tumor metabolic burden, have shown that these measures are either more accurate than SUV_max_ or the sole prognostic marker of outcome in NSCLC (13–15). Therefore, MTV and TLG may potentially be better surrogate imaging markers of tumor biology than SUV_max_.

The present study showed that the occurrence rate of BM was associated with adenocarcinoma (HR=5.715; *p*=0.024) according to the univariate analysis. Similar results have been shown in several studies, in which patients with adenocarcinoma or nonsquamous cell carcinoma had a significantly higher incidence of developing BM than squamous cell carcinoma, with a relative risk in the range of 2.1 to 4.1 (25–27). This may be related to the pathological and biological characteristics that the adenocarcinoma subtype is generally more invasive than the squamous cell carcinoma subtype (28).

The current study had some limitations. First, the study design was retrospective and we had a small sample size, and absence of an independent set of patients to perform verification. Furthermore, the heterogeneous treatment protocols may have a confounding effect on prognostication. Further prospective studies with larger homogeneous patient cohorts are warranted to validate the results. Second, due to the limited number of patients enrolled, we did not analyze whether there were differences in PET parameters between patients with BM and other distant metastasis. More patient cohorts were needed to analyze these differences in the next study. However, our results preliminarily indicate that baseline metabolic tumor burden can provide predictive information for BM development. Third, the accuracy of the metabolic tumor burden may be affected by the false-negative and false-positive results of ^18^F-FDG PET. In this study, we try to reduce such deviation by reading ^18^F-FDG PET together with diagnostic CT.

## Conclusions

Baseline metabolic tumor burden at the level of the metastatic lymph nodes and whole-body tumors as measured with MTV and TLG on ^18^F-FDG PET are significant predictive factors for BM development independent of T and N stages and are better predictive imaging biomarkers than SUV_max_ in patients with locally advanced NSCLC. Therefore, the addition of tumor burden measurements can help further stratify patients within each stage and optimize treatment methods. These results will need to be validated in a multicenter prospective study with larger homogeneous patient cohorts.

## Data availability statement

The original contributions presented in the study are included in the article/Supplementary Material. Further inquiries can be directed to the corresponding authors.

## Ethics statement

The studies involving human participants were reviewed and approved by The institutional review board of the First Affiliated Hospital of Jinan University. Written informed consent for participation was not required for this study in accordance with the national legislation and the institutional requirements. Written informed consent was not obtained from the individual(s) for the publication of any potentially identifiable images or data included in this article.

## Author contributions

JS and HY contributed to the writing of the manuscript. JS, HY, CD, YL and YC collected the original data. JS contributed to the statistical analysis of the data and constructed the figures and tables. JS, YT, BG and JG contributed to the analysis of imaging information. XL and HX conceived the paper layout and modified the paper. All authors had read and approved the final version of the manuscript.

## Funding

This work was financially supported by the Fundamental Research Funds for the Central Universities (21619352) and Funding by Science and Technology Projects in Guangzhou (202201010168).

## Acknowledgments

We would like to thank the staff members of the Department of Nuclear Medicine and PET/CT-MR Centre, First Affiliated Hospital of Jinan University, for their excellent technical support.

## Conflict of interest

The authors declare that the research was conducted in the absence of any commercial or financial relationships that could be construed as a potential conflict of interest.

## Publisher’s note

All claims expressed in this article are solely those of the authors and do not necessarily represent those of their affiliated organizations, or those of the publisher, the editors and the reviewers. Any product that may be evaluated in this article, or claim that may be made by its manufacturer, is not guaranteed or endorsed by the publisher.
